# The Mentorship Blueprint: A Comprehensive Review for the Development of Programs in Pharmacy Education

**DOI:** 10.3390/pharmacy13010029

**Published:** 2025-02-18

**Authors:** Jason W. Guy, Shelby Smart, Mollie Harber, Julie H. Oestreich

**Affiliations:** College of Pharmacy, University of Findlay, Findlay, OH 45840, USAjulie.oestreich@findlay.edu (J.H.O.)

**Keywords:** mentorship programs, pharmacy mentorship, student mentorship, faculty mentorship, mentorship resources

## Abstract

**Background:** Mentorship has benefits for students and faculty, helping to support their professional development, connectedness, and career endeavors. While the value of mentorship programs is well documented in the literature, there is less practical guidance and few compiled resources to start a program. This paper reviews different mentorship practices in pharmacy education and provides a list of strategies to develop high-functioning mentorship programs or groups. **Methods:** A review of the literature was conducted through PubMed and other databases. If the titles and abstracts met the initial criteria for relevance to the topic, the complete article was reviewed in the context of the inclusion and exclusion criteria. Included articles focused on mentorship, mentorship programs, mentorship development, mentoring faculty or students, or mentoring in the workplace. **Results:** Twenty-three studies were included in the final review. Summaries and key points from the studies were reviewed and discussed. The advantages of mentorship programs include increased social connection, goal setting, and professional development. Challenges include increased time commitments and difficulty in determining objective markers of success. Critical components have been extracted from the literature, and key resources and templates have been provided to aid in mentorship program development. **Conclusions:** This review summarizes the pharmacy mentorship literature and provides user-friendly tables to quickly locate resources to build a mentorship program in pharmacy education.

## 1. Introduction

Mentorship has been defined as “a professional, working alliance in which individuals work together over time to support the personal and professional growth, development, and success of the relational partners through the provision of career and psychosocial support” [1]. Mentorship helps future generations of students and faculty to flourish in their profession. Studies have shown that effective mentorship results in growth for both students and faculty in their careers [2,3,4].

Supporting and developing quality employees through enhanced professional development is critical for institutions to perform at their peak. Offering this development through mentorship allows for learning in both directions (mentor to mentee and mentee to mentor) as both parties gain new perspectives from each other. While many studies are available that describe mentorship programs, a succinct summary and practical guide with examples to develop a full mentorship program would be beneficial [5].

Mentorship can be provided in a variety of formats and can range from personalized one-on-one mentorship to mentorship groups of various sizes with multiple mentors or mentees [6]. One-on-one mentorship can include random assignments in some programs, or the process could be highly personalized based on established relationships or the completion of personality assessments. In addition, mentorship offerings can range from in person to remote, and meetings can vary in terms of frequency based on the preferences of the mentor and mentee [7]. Generally, the mentor is a seasoned veteran who can help more junior colleagues in terms of career and life goals, although other mentorship models use a near-peer or layered learning approach where a more senior faculty/student or trainee mentors a junior faculty/student or trainee [8].

Individual mentor–mentee pairings (or dyads) have a long history and help to achieve goals with a simple design. Most research is focused on the mentor–mentee relationship; however, new formats of mentoring have appeared more frequently in the last few decades [1]. Group mentoring (or triad mentorship) provides multiple vantage points and perspectives for a mentee and allows a layered approach through multiple levels of mentorship. A triad approach allows for increased synergy and networking but can be difficult to schedule and may create competition or adverse relationship dynamics within the group [9].

In pharmacy education, mentorship typically occurs between new faculty and experienced faculty, between faculty and students, or through student-to-student peer mentoring. For example, faculty applying for tenure and/or promotion may leverage mentorship to help to develop goals and timelines to achieve their desired outcomes [6,10]. More experienced faculty also benefit from mentorship when moving into new leadership positions [6]. Offering mentorship support for faculty at all levels can help retention, as well as enhancing skills that lead to more productive teaching and research [6]. Indeed, previous research has shown an increase in scholarly activity and grant funding for faculty who received mentorship [6]. Mentorship programs also strengthen collegiality and positive cultures in the workplace [6], leading to enhanced relationships linked to positive well-being and career outcomes [11,12].

From a student perspective, mentorship offers opportunities to learn from pharmacists or others who are completing a pharmacy program. As mentioned before, mentorship can enhance well-being and provides an opportunity for students to network with faculty or their peers, which could improve their job prospects in the future. Some research has shown that mentorship increases communication, leadership, and professionalism, which are all important components of a student’s development into a pharmacist [2,13]. Additionally, research suggests that mentorship can improve academic success [14].

From the institution perspective, declining enrollment at many colleges has made developing relationships with students especially important as a means to increase belonging and retention [15]. Successful mentorship models support social connection through peer-to-peer or faculty-to-student engagement [16,17,18] and serve as an opportunity to enhance retention through improved well-being and social connection.

Notwithstanding the benefits for individuals and institutions, developing a mentorship program can be daunting. Fortunately, a helpful checklist highlights the key features for the development of a faculty mentorship program [5]. We expand on this work by adding the literature on student mentorship programs and collating example templates and summary information [5]. In addition to reviewing the different mentorship practices in pharmacy education, this paper outlines evidence-based strategies to develop high-functioning mentorship programs for students and faculty.

## 2. Materials and Methods

A comprehensive literature review was conducted using Google Scholar, EBSCOhost (via the University of Findlay Library), and PubMed. These databases were selected for their extensive coverage of research across academic disciplines. The search terms utilized were mentoring, education, pharmacy, mentors, students, faculty, academic success, academic failure, mentorship, college, higher education, doctorate programs, mentorship program development, mentorship programs, academic achievement, and mentoring practices.

If the titles and abstracts met the initial criteria for relevance to the topic, the complete article was reviewed in the context of the inclusion and exclusion criteria. Specifically, the article had to include pharmacy-related information about mentorship, mentorship programs, mentorship development, mentoring faculty or students, or mentoring in the workplace. Articles were excluded if they included students in postgraduate PhD/residency/fellowship training, clinical settings, or mentorship in other disciplines besides pharmacy. No timeframe restrictions were used, and all duplicate articles were removed. Categorization was completed based on the article focus, and descriptive counts of the number of articles included in each category were determined.

## 3. Results

The initial search yielded 1630 articles, and 23 articles met the criteria for inclusion in the study—13 focused on student mentorship (Table 1) and 10 focused on faculty mentorship (Table 2). Setting goals and expectations was cited as an important recommendation when establishing relationships between mentors and mentees. Additionally, several studies identified concerns about the time commitment needed and the unsuccessful pairing of some mentors and mentees. Many of the student-focused studies showed benefits, either through improved performance or student satisfaction. Table 2 highlights the references and corresponding descriptions for faculty mentorship programs in pharmacy. Many studies indicated that faculty were satisfied with the program offerings, although logistical hurdles and scheduling served as barriers. Overall, most participants liked the social connection established. Defining expectations early in the process and offering training for mentors were viewed as important components of mentoring programs.

## 4. Discussion

Mentoring confers several benefits to its participants, including enhanced professional skills, structured networking, and improved markers of achievement [16]. Indeed, Jackevicius et al. reported that 96.4% of mentees felt that a mentorship program aided in their success as a faculty member [28]. Additionally, 86% of students in one study found mentorship beneficial [18], although other studies did not reach this level of agreement. Many articles support the idea of mentorship to combat social isolation, although challenges should also be considered when developing a new program and recruiting mentors and mentees (Table 3).

Table 4 highlights the critical components and key themes identified in our review, including (1) formal training for mentors (2) the need for authentic communication and engagement through setting clear goals and expectations, (3) the careful selection of teams, and (4) appropriate input from institutional stakeholders to tailor the mentorship program design. Since mentors are less likely to perceive a benefit from the relationship [18], it is especially critical to highlight the value of serving in this role and the opportunity to serve others and learn something new. Additional financial or time incentives may also be helpful [31]. Mentees, on the other hand, are more likely to recognize mentorship as an opportunity to grow professionally and improve their chances for career success. To maximize the mentorship process, it is imperative to thoughtfully match the mentors and mentees to align their personalities and communication preferences. Goal setting is also important at the mentor and mentee level to ensure that expectations are aligned and progress is made. Mentorship teams should take time to write down shared goals and regularly evaluate them to stay on track. Several resources mentioned an additional program component—the necessity to train mentors so that they are well prepared to guide mentees and offer support. This training could take several forms, from informal to formal, but some training or resource guide is essential to support mentors and establish clear expectations for their role [4,5,17]. When starting a mentorship program, institutional leaders should consider the end goals of the program and establish appropriate metrics for the evaluation of success [30]. Table 4 lists example resource kits to help to train mentors, set expectations, and choose the best mentorship style for a program [4,5].

### 4.1. Practical Application

Establishing a new mentorship program can be a large task, but taking time to reflect on the intended mission and key aspects of the program while learning from the experiences of others may increase the likelihood of success [34]. As outlined in Table 4, the collective literature on pharmacy mentorship programs suggests the following features. First, determine the purpose of the program and define the structure and participants. Evidence is conflicting regarding whether one-on-one or group mentoring is most effective, which leaves flexibility for the institution to meet the needs and preferences of the stakeholders [1].

The next critical step is to invite participants and implement a survey to assess the specific goals and outcomes desired by these participants. Determining who will participate in the mentorship program and how they will be selected is an important step, and the literature suggests that taking the time to understand what each individual is hoping to achieve will result in higher rates of program success [30]. Some research also supports stipends, protected time, or other incentives to encourage active mentor involvement [31]. Programs can assess additional factors like personality types and communication styles through surveys, as highlighted in Table 4. These available tools could be modified to meet institution-specific needs. During this step, it is also critical to train mentors and mentees and set shared expectations for communication and the overall program (see Table 4 for examples of goal setting documents and training) [5,16,30].

Finally, the developers of a mentorship program should formally evaluate its effectiveness and assess whether programmatic and individual goals were met. Participants should be surveyed on their perceptions of the program and what quality improvements should be made for future iterations. Defined metrics for evaluation that align with the goals of both the mentors and mentees are important to ensure satisfaction. Some relevant metrics identified in the literature include career advancement, satisfaction with the overall mentorship, the quality of the relationship, progress in building resilience, the number and quality of activities performed, retention, and the achievement of the stated personalized goals [4,5,13,18,20]. Additional markers for success may include how well an individual adjusts to a new institution or leadership position [26]. This could be measured by how quickly they become independent in their new role [26]. The consistent use of formal surveys and evaluation tools also allows for data comparison to evaluate changes and successes over multiple iterations of the program [4].

Several barriers exist when starting a mentorship program, including the most obvious—the significant time commitment for mentors but also mentees [13]. Some strategies may mitigate this to an extent, such as creating agendas for each meeting to maintain focus and matching mentorship teams with similar preferences regarding meeting times and frequencies. Using technology like meeting scheduler tools or remote conferencing platforms to help with the scheduling logistics can also be beneficial [17]. This challenge also further supports the recommendation to provide incentives for mentor involvement [31].

### 4.2. Limitations

This review and blueprint for mentorship programs possesses several notable limitations. First, we limited the eligibility to the pharmacy education literature, which excluded results from valuable mentorship programs in other fields.

Second, having tangible end products or markers can be difficult to measure with mentoring activities. Therefore, it is important to evaluate programmatic goals after each program offering to ensure that mentorship programs fulfil their purpose [18,29]. This is especially useful if a concrete marker like a faculty promotion or student postgraduate placement is not relevant.

### 4.3. Key Takeaways

- Mentorship supports connectedness and professional growth for both mentees and mentors.
- Critical factors like setting goals, providing training, choosing teams thoughtfully, and assessing the effectiveness of mentorship relationships are all important in providing a quality mentorship program.
- Challenges in implementing a mentorship program include time commitments, scheduling, poor communication between mentors and mentees, and mismatched goals.
- Many resources and templates already exist to support mentorship program development.

## 5. Conclusions

This paper provides an in-depth review of both student- and faculty-centered mentorship programs in pharmacy education. Mentorship improves satisfaction and career advancement, especially when the mentoring program is supported by structured training and expectation/goal setting. This review summarizes the pharmacy mentorship literature and provides a list of resources to help to build a quality mentorship program for students or faculty.

## Figures and Tables

**Table 1:** Mentoring programs focused on students.

Mentoring Focus	Summary of Results	Key Takeaways
**References that Evaluated Student Growth and Development**		
Pharmacist-to-student mentorship Rahal et al. [2]	Clinical mentors helped students to develop communication and leadership skills, recognize career opportunities, manage challenges, implement theoretical knowledge, and interpret constructive feedback.	Students who experienced mentoring felt motivated to learn, inspired to follow their goals, and an improved sense of well-being.
Resident-to-student mentorship Nisly et al. [8]	Students rated their resident mentors as knowledgeable, stating that they provided valuable feedback.	This mentorship program allowed residents to enhance their mentorship abilities while students received feedback on their presentations (patient case scenarios).
Pharmacist-to-student mentorship Waghel et al. [13]	A practitioner-to-student mentoring program was well received. Discussions on interviewing and career stories were activities that were well received. Time limitations and scheduling were the most cited challenges.	Providing stories of career experiences from practitioners can be beneficial to students. This provides another avenue for experiential learning.
Lecturer-to-student mentorship Jegede et al. [14]	Mentor relationships with lecturers improved the academic performance, study habits, and self-confidence of students.	Mentorship with lecturers benefits students academically through role modeling and support.
Peer-to-peer student mentorship Raub et al. [19]	In one program, 77% of students felt that mentorship aided in their professional growth, and 76% of senior mentors believed that having a previous student mentor aided in becoming efficient mentors themselves.	A peer mentorship program may aid in student growth and development.
Peer-to-peer student mentorship Etzel et al. [20]	The majority of respondents (71%) thought that the program positively aided in the transition to pharmacy school, although no significant improvement in retention was found.	Mentorship programs may provide a benefit for students transitioning into pharmacy programs.
Faculty-to-student mentorship Lahiri et al. [21]	Both students and faculty perceived benefits from the structured faculty advising program—faculty felt more engaged and students felt more supported, resulting in a 30% improvement in advising satisfaction.	The majority of participants appreciated the value of the revised advising program and meeting opportunities to build relationships and long-term connections.
Peer-to-peer student mentorship Sin et al. [22]	The peer mentoring program improved satisfaction and engagement.	When students are effectively paired together in a mentoring program, it allows for growth personally, professionally, and academically.
Faculty-to-student mentorship Arya et al. [23]	Preceptor involvement can be critical to student development, especially in a mentorship role. It is important to provide feedback to students, as well as understand the expectations of the student and institution.	Students need feedback for growth. Setting goals and expectations can help both the mentor and mentee to grow as part of mentorship.
Peer-to-peer student mentorship Brown et al. [24]	In this program, 74% of the mentees and 64% of the mentors had a positive experience.	Pairing students based on gender and similar hobbies can help a mentorship program to succeed.
**References that Focused on Developing a Mentorship Program**		
Peer-to-peer student mentorship Asal et al. [16]	This study highlights several elements of mentorship based on 3 separate programs at colleges of pharmacy. Major elements found were selecting mentors and mentees based on surveys and providing mentorship coaching and support.	Mentor support is needed to help facilitate successful pairings. Utilizing surveys to find good matching between individuals is helpful.
Faculty-to-student mentorship Rowe et al. [18]	More students than faculty mentors (86% to 62%). benefitted from a mentorship program focused on setting goals, fostering resilience, and career exploration. Ineffective matching of mentor–mentee pairs and communication were cited as challenges.	Shared goal setting is key to developing successful faculty–student relationships in mentorship programs.
Faculty-to-student mentorship Witry et al. [25]	Focus groups identified that mentees held high expectations for the mentors (engaged, similar interests, etc.), even though time constraints hindered these interactions.	Establishing expectations for mentors and mentees is critical for the success of the program.

**Table 2:** Mentoring programs focused on faculty.

Author	Results	Discussion
**Faculty-Focused References—Faculty Development**		
Eiland et al. [3]	In a team-mentoring program for junior faculty, 94% of mentees found the teams helpful, and 90% requested no changes to their mentor team. Importantly, 75% of participants stated that the mentorship approach helped them to achieve a promotion.	The top areas in which junior faculty stated that they improved with assistance from the mentor teams were APPEs (clinical teaching), promotion, and scholarship.
Hennessey et al. [26]	A mentoring program designed to help faculty to become course coordinators led to a higher mean course grade and student evaluation scores compared to the prior five years.	This program allowed for a quicker and easier transition for faculty to become course coordinators.
Eiland et al. [27]	Most participants were satisfied with a mentorship program organized by a professional organization (AACP). Career goal development, work/life integration, and difficult work situations were the most frequently discussed topics.	This nationwide mentoring program for pharmacy faculty allowed mentees to address areas such as career development and research. A unique feature was utilizing a professional organization as a platform for mentorship, rather than home institutions.
Jackevicius et al. [28]	In one program, 96.4% of faculty felt that mentorship developed their abilities and aided in their success as faculty.	The majority of mentors provided insights into time management, what to prioritize, and how to create work/life balance.
**References Focused on Development of Faculty Mentorship Programs**		
Metzger et al. [4]	In this program, an overview of mentor characteristics helped participants to understand the matching process and expectations. Assessment examples were provided to evaluate mentees, mentors, and the overall program.	In creating a new faculty mentorship program, an institution must consider its mission, resources (from a cost and workload perspective), and size.
Law et al. [5]	Checklists help to form a structured mentorship approach and may include aspects such as mentorship pairing, training, resources, and evaluation.	Successful mentoring requires flexibility, mentor training, and regular assessment.
Biehle et al. [17]	The women involved in a peer mentoring circle enjoyed the group, finding support, mentorship, and a safe sounding board to discuss issues related to their professional lives. The circle created topic lists for each monthly meeting. Challenges included scheduling, time commitments, online format logistics, and efforts needed to develop trust.	Recommendations for the creation of a peer mentoring circle included having gender-exclusive circles, selecting members from different institutions, establishing trust in the circle, and allowing mentees to specify the topics and their needs.
Kinney et al. [29]	One study found high percentages of satisfaction for mentees (80%) and mentors (86%).	There is no single method of structuring a mentorship program. Some qualities of a successful program included clearly defined goals, defined program outcomes, and the support of the organizing institution.
Shields et al. [30]	A review of AACP affinity groups found that a focus on both mentor and mentee needs is critical for success. Mentorship connections provide sources of support and empathy. Preferences in matching were also used.	Mentor programs often fall short in creating a foundation of trust and authenticity in the matched pair if they do not account for the needs and goals of the mentee (e.g., help with promotion, job responsibilities, work/life balance, etc.). Setting expectations, training mentors and mentees, and aligning goals are critical for success.
Minshew et al. [31]	In this program, strengths included the structure and relationships developed. Challenges included time and mentor–mentee matching, even though the 2014 Pairs checklist [5] was utilized.	The program strengths coincided with the AACP Joint Council Task Force on Mentoring recommendations. Resources must be provided to ensure a successful program, particularly incentives for the mentors, such as protected time or stipends.

**Table 3:** Advantages and challenges with mentorship programs.

Advantages	Challenges
Improved markers of success (promotion, etc.) [3,6,10]	Time commitment and scheduling [31]
Professional development for all participants [2,13]	Mismatched goals, expectations, or personalities [32]
Possible increased retention of both students and faculty through increased relationship building [33]	Mentoring skills take time to develop [34]
Improved well-being and social connection [16,17,18,33]	Markers for success may be difficult to measure

**Table 4:** Critical components and example resources for mentorship programs.

Critical Component	Guidance and Resources
Define characteristics of the mentorship program [31,35] See the references below for example mentoring programs: Reference [31] Reference [35]	Define group size or one-on-one mentoring (see reference [35] for menu of options) Secure stipends or protected time to incentivize mentors (see reference [31])
Carefully select mentorship pairings or teams Consider a survey to match individuals [16,18,36,37,38] See the references below for example surveys and worksheets to help in selecting mentor-mentee pairs: Reference [18] Reference [36] Reference [37] Reference [38]	- Incorporate assessments of personality types or communication styles to ensure better matching. Provide templates to optimally match the goals and passions of both mentors and mentees. Templates in the literature include the following Surveys to match mentors and mentees (see reference [36]); Mentor and mentee self-assessments (see references [37,38]).
Provide training to mentors and mentees [5,16,30,31,39,40,41,42,43,44] See the references below: Reference [39] Reference [40] Reference [41] Reference [42] Reference [43] Reference [44]	- Provide a guidebook and training at the beginning of the mentorship program (see references [39,40,41,42,43,44] for examples) - Provide articles focused on mentorship, including the following: Example questions to start the process of mentorship (see reference [40]); A list of tips for effective mentorship (see references [39,42]).
Set goals at beginning of program to establish expectations [4,30,45,46,47,48,49] See the references below for examples of expectation and goal setting documents: Reference [46] Reference [47] Reference [48]	- Program level goals should include intended outcomes and an evaluation plan (see references [46,47,48] for example SMART Goals) - Mentoring pairs or teams should ensure the following: Communicate on the frequency of meetings and any special scheduling needs; Develop a list of topics for discussion throughout the program (see reference [47]); Set an agenda for each meeting; Establish SMART goals for intended outcomes and agree on measurements for success (see reference [48]).

## Data Availability

Data are contained within the article.
